# Supplementary material for: Searching for universal model of amyloid signaling motifs using probabilistic context-free grammars
Source: BMC Bioinformatics. 2021 Apr 29;22:222. doi: 10.1186/s12859-021-04139-y (PMC8086366; doi:10.1186/s12859-021-04139-y)
Supplement: Supplementary file 2 — Additional file 2. Figure S1: The outline of the processing pipeline. [file 12859_2021_4139_MOESM2_ESM.pdf]

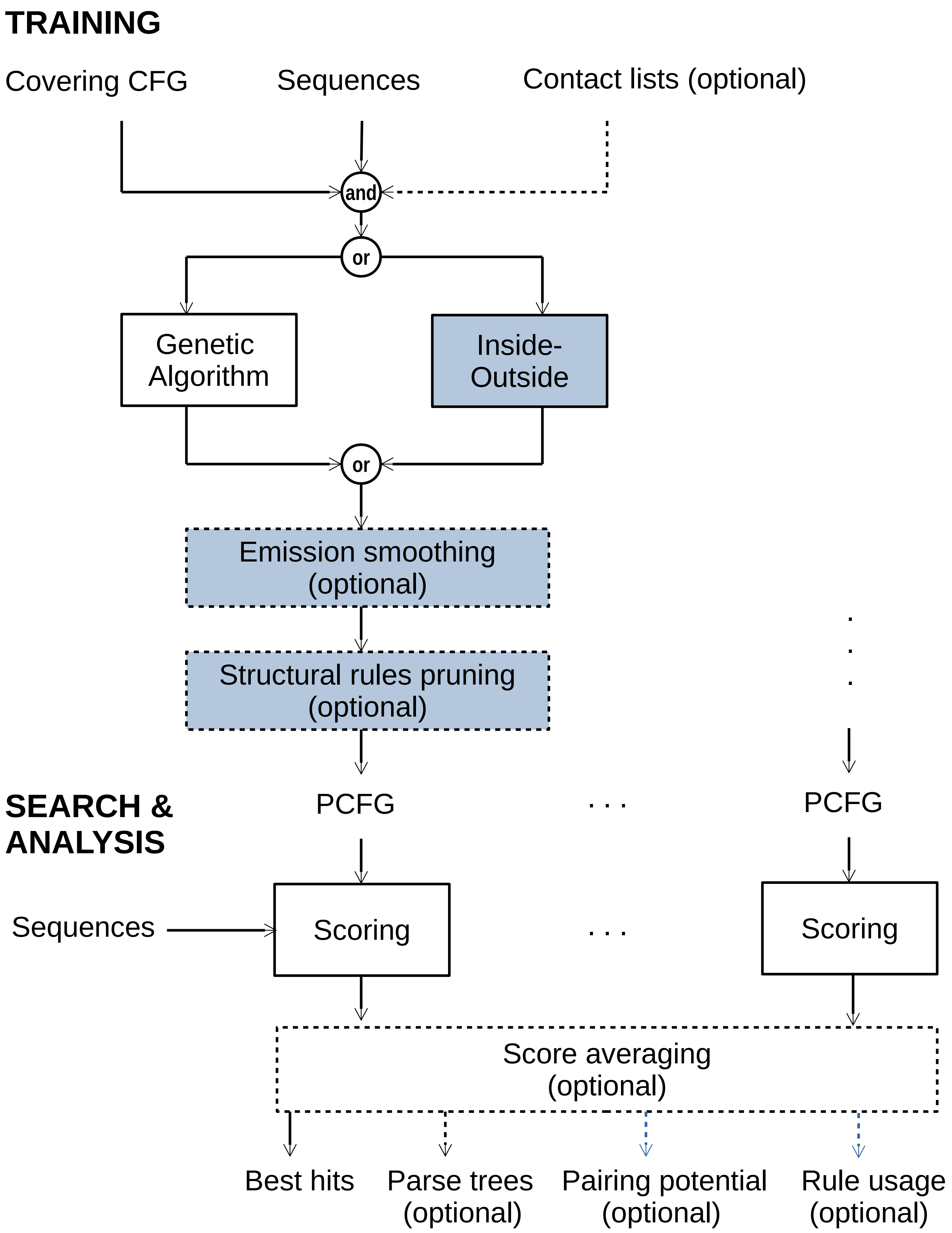

Supplementary Figure 1: The outline of the processing pipeline in the probabilistic context-free grammar-based framework for protein sequence analysis. Optional features, inputs and outputs are marked with dotted lines. Newly introduced features and outputs are marked with grey blue.
