# Supplementary material for: Searching for universal model of amyloid signaling motifs using probabilistic context-free grammars
Source: BMC Bioinformatics. 2021 Apr 29;22:222. doi: 10.1186/s12859-021-04139-y (PMC8086366; doi:10.1186/s12859-021-04139-y)
Supplement: Supplementary file 3 — Additional file 3. Table S1: Analytical data for peptide synthesis and purification. [file 12859_2021_4139_MOESM3_ESM.pdf]

Name	Full name	Formula	$M_{cal.}$[1]	M_{MS}[2]	HPLC $t_{ret.}$[3] [min]
PPT_1	ORT49035.1_103_123	$C_{98}H_{163}N_{33}O_{31}$	2300.2<br>[1/2M+1] 1150.62<br>[1/3M+1] 767.42<br>[1/4M+1] 575.81	[1/2M+1] 1150.62<br>[1/3M+1] 767.06<br>[1/4M+1] 576.05	8.840
PPT_4	SesB_349_385	$C_{168}H_{246}N_{50}O_{59}$	3910.83<br>[1/2M+1] 1955.90<br>[1/3M+1] 1304.27<br>[1/4M+1] 978.46	[1/2M+1] 1955.94<br>[1/3M+1] 1304.27<br>[1/4M+1] 978.43	8.350
PPT_5	AEB69175.1 5 29	$C_{117}H_{197}N_{39}O_{36}$	2726.8<br>[1/2M+1] 1363.75<br>[1/3M+1] 909.50<br>[1/4M+1] 682.38	[1/2M+1] 1363.75<br>[1/3M+1] 909.50<br>[1/4M+1] 682.38	7.763
PPT_6	RDW70414_382_421	$C_{146}H_{232}N_{58}O_{63}$	3808.6<br>[1/2M+1] 1904.85<br>[1/3M+1] 1270.23<br>[1/4M+1] 952.93	[1/2M+1] 1904.98<br>[1/3M+1] 1270.24<br>[1/4M+1] 952.93	7.841

[1] $M_{cal.}$ – calculated mass of the peptide
[2] M_{MS} – found mass of the peptide using HRMS
[3] HPLC $t_{ret.}$ – retention time in analytical HPLC spectra
