# Supplementary material for: Searching for universal model of amyloid signaling motifs using probabilistic context-free grammars
Source: BMC Bioinformatics. 2021 Apr 29;22:222. doi: 10.1186/s12859-021-04139-y (PMC8086366; doi:10.1186/s12859-021-04139-y)
Supplement: Supplementary file 4 — Additional file 4. Figure S2: Representative light microscope images of the peptides stained with CR. [file 12859_2021_4139_MOESM4_ESM.pdf]

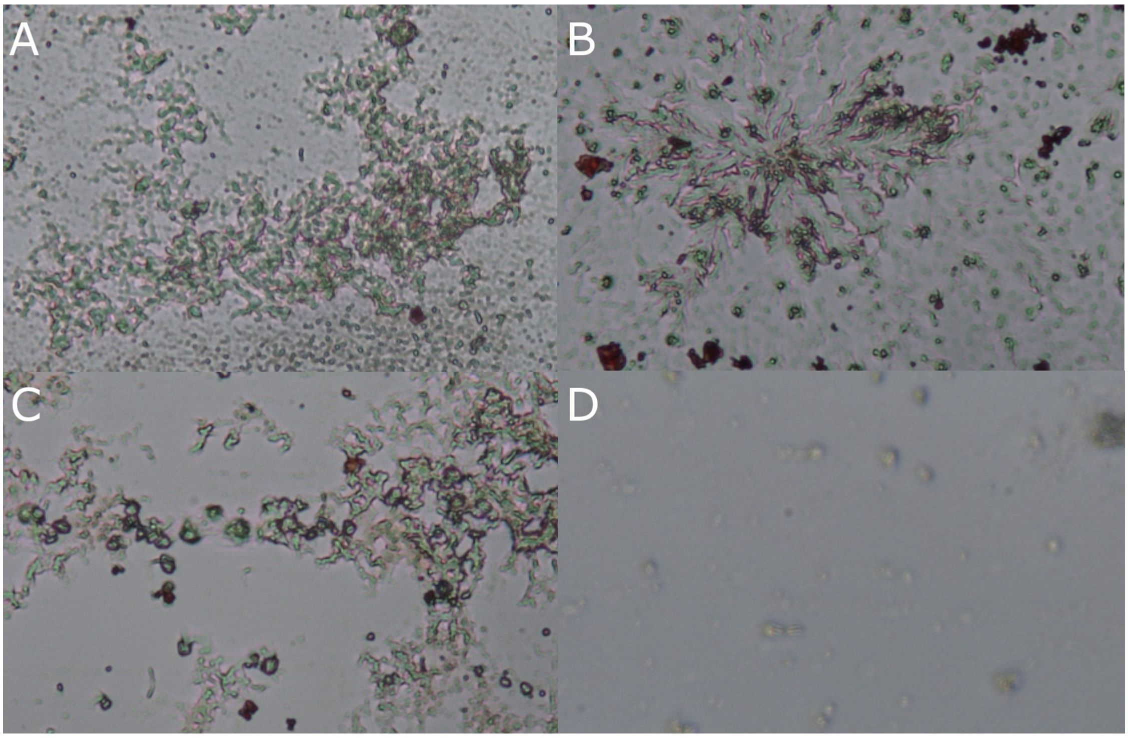

Supplementary Figure 2: Representative light microscope images of the peptides stained with Congo Red. Original magnification x400. (**A**) ORT49035_103_123, (**B**) AAS80314.1_349_385, (**C**): AEB69175_5_29, and (**D**): RDW70414_382_421. C_{pep} = 50 µM
