# Supplementary material for: Searching for universal model of amyloid signaling motifs using probabilistic context-free grammars
Source: BMC Bioinformatics. 2021 Apr 29;22:222. doi: 10.1186/s12859-021-04139-y (PMC8086366; doi:10.1186/s12859-021-04139-y)
Supplement: Supplementary file 5 — Additional file 5. Table S2: Experimental conditions for the ATR-FTIR spectroscopy and CR staining. [file 12859_2021_4139_MOESM5_ESM.pdf]

Supplementary Table 2: MIRRAGGE – Minimum Information Required for Reproducible AGGregation Experiments

**Sample details**						
No.	Organism/Peptide Sequence	UniProt code (residues)	pI	GRAVY	Extinction coefficient [A280, 0.1% (w/v)]*	MW from chemical composition (Da)
**ORT49035_103_123**	**VDLRDAKGVQVGDGNVQINRF**	A0A1X1MP51 (103-123)	1	-0.42	68500	2299.5
**AAS80314_349_385**	**SFNNLGSGDQFNTPGGTQNINKGGNEV SGGNFYGSVQF**	Q6PX79 (349-385)	6.64	-0.77	147340	3910.05
**AEB69175_5_29**	**KSRFDQRGQKVIGQQINVAGDATLP**	F4C0B0 (5-29)	11.39	-0.72	80020	2726.06
**RDW70414_382_421**	**GAPANNTSNSIQHNNSGSGHQNSGSGQ QNIGTNTGSGQQ**	A0A3D8R976 (382-421)	1.2	-1.42	121640	3807.76

Item	Parameter	Sub-parameter	Value
Source (supplier, catalogue No. or reference)			"in house"
N-terminal modification			----
C-terminal modification			----
Internal modifications			----
Other modifcations			----
Purity (%)			≥95%
**Purification** (If applicable)	Chromatography techniques		RP-HPLC
	Concentration of stock solution (M, mg/mL)		4 mg/mL
	Storage/Reconstitution buffer		water/ACN
	Method of protein quantification		UV/VIS
	Storage conditions		Lyophilized
	Additional key information		----
**Sample quality control**			
**Polishing step**	Immediately before the aggregation assay		----
	Concentration (M, mg/mL)		----
	Method quantification		----
**Aggregation assay**	Method of detection		ATR-FTIR; **CR**
	Equipment details		Nicolet 6700 spectrometer (Thermo Scientific, USA) equipped with ATR Accessory with Heated Diamond Top-plate (PIKE Technologies, USA); **ECLIPSE 50i (Nikon, Japan)**
	Measurement parameters		128 scans, 4 cm^{-1}, T= 25 ºC;
	Plate/cuvette reference		-----
	Assay volume		10 µL
	Evaporation control method		----
	Seeding details (if applicable)		----
	Shaking	Intensity	----
		Shaking mode	----
		Frequency	----
	Beads	Reference	----
		Number/ assay	----
	Temperature (ºC)		37
	Concentration (M, mg/mL)		4 mg/mL; **10 µg/mL**
	Aggregation buffer and additives		D_2O
	Measurement frequency		----
	Assay duration		2h
	Plate/cuvette setup		----
	Additional key steps		----

* Calculated based on: http://bestsel.elte.hu/extcoeff.php [Extinction coefficient at 205 nm, concentration units: $M^{-1}cm^{-1}$
